# Behavioral and Biochemical Insights into the Therapeutic Potential of Mitocurcumin in a Zebrafish–Pentylenetetrazole (PTZ) Epilepsy Model

**DOI:** 10.3390/ph18030382

**Published:** 2025-03-07

**Authors:** Alin Dumitru Ciubotaru, Carmen-Ecaterina Leferman, Bogdan-Emilian Ignat, Anton Knieling, Irina Mihaela Esanu, Delia Lidia Salaru, Liliana Georgeta Foia, Bogdan Minea, Luminita Diana Hritcu, Cristina Daniela Dimitriu, Laura Stoica, Ioan-Adrian Ciureanu, Alin Stelian Ciobica, Andrei Neamtu, Bogdan Alexandru Stoica, Cristina Mihaela Ghiciuc

**Affiliations:** 1 Discipline of Pharmacology, Faculty of Medicine, Grigore T. Popa University of Medicine and Pharmacy, 16 Universitatii Street, 700115 Iasi, Romania; alin-dumitru_ciubotaru@umfiasi.ro (A.D.C.); carmen-ecaterina.leferman@umfiasi.ro (C.-E.L.); cristina.ghiciuc@umfiasi.ro (C.M.G.); 2 Discipline of Biochemistry, Faculty of Medicine, Grigore T. Popa University of Medicine and Pharmacy, 16 Universitatii Street, 700115 Iasi, Romania; daniela.dimitriu@umfiasi.ro; 3 Discipline of Neurology, Faculty of Medicine, Grigore T. Popa University of Medicine and Pharmacy, 16 Universitatii Street, 700115 Iasi, Romania; emilian.ignat@umfiasi.ro; 4 Neurology Department, Clinical Rehabilitation Hospital, 14 Pantelimon Halipa Street, 700661 Iasi, Romania; 5 Discipline of Forensic Medicine, Faculty of Medicine, Grigore T. Popa University of Medicine and Pharmacy, 16 Universitatii Street, 700115 Iasi, Romania; anton.knieling@umfiasi.ro; 6 Institute of Forensic Medicine, 4 Buna Vestire Street, 700455 Iasi, Romania; 7 Department of Medical Specialties I, Faculty of Medicine, Grigore T. Popa University of Medicine and Pharmacy, 16 Universitatii Street, 700111 Iasi, Romania; irina.esanu@umfiasi.ro; 8 Institute of Cardiovascular Diseases, 50 Carol I Avenue, 700503 Iasi, Romania; deliasalaru@gmail.com; 9 Discipline of Biochemistry, Faculty of Dental Medicine, Grigore T. Popa University of Medicine and Pharmacy, 16 Universitatii Street, 700115 Iasi, Romania; georgeta.foia@umfiasi.ro (L.G.F.); bogdan-minea@umfiasi.ro (B.M.); 10 Internal Medicine Clinic, Ion Ionescu de la Brad University of Life Sciences, 3 Mihail Sadoveanu Alley, 700490 Iasi, Romania; lumidih@yahoo.com; 11 Discipline of Cell and Molecular Biology, Grigore T. Popa University of Medicine and Pharmacy, 16 Universitatii Street, 700115 Iasi, Romania; laurastoica2004@yahoo.com; 12 Department of Medical Informatics and Biostatistics, Grigore T. Popa University of Medicine and Pharmacy, 16 Universitatii Street, 700115 Iasi, Romania; adrian.ciureanu@umfiasi.ro; 13 Department of Biology, Faculty of Biology, Alexandru Ioan Cuza University of Iasi, 20A Carol I Avenue, 700505 Iasi, Romania; alin.ciobica@uaic.ro; 14 Center of Biomedical Research, Romanian Academy, Iasi Branch, 2 Teodor Codrescu Street, 700481 Iasi, Romania; 15 “Ioan Haulica” Institute, Apollonia University, 11 Păcurari Street, 700511 Iasi, Romania; 16 Academy of Romanian Scientists, 54 Splaiul Independentei, 050094 Bucharest, Romania; 17 Department of Physiology, Grigore T. Popa University of Medicine and Pharmacy, 16 Universitatii Street, 700115 Iasi, Romania; andrei.neamtu@umfiasi.ro; 18 St. Mary’s Emergency Children Hospital, 62 Vasile Lupu Street, 700309 Iasi, Romania

**Keywords:** epilepsy, oxidative stress, zebrafish model, curcumin, sodium valproate, mitocurcumin, mitochondria-targeted antioxidants, pentylenetetrazole, behavioral analysis

## Abstract

**Background/Objectives**: Epilepsy is a complex neurological disorder with a strong link to oxidative stress, which contributes to seizure susceptibility and neuronal damage. This study aims to investigate the effects of curcumin (Cur), sodium valproate (VPA), and mitocurcumin (MitoCur), a mitochondria-targeted curcumin, on behavioral and oxidative stress parameters in a zebrafish model of pentylenetetrazole (PTZ)-induced seizures. **Methods:** Adult zebrafish were exposed to two concentrations (0.25 and 0.5 µM for Cur and MitoCur; 0.25 and 0.5 mM for VPA). Behavioral assessments, including locomotion, spatial exploration, and directional movement, were conducted using EthoVision XT tracking software. Oxidative stress markers, including superoxide dismutase (SOD), malondialdehyde (MDA), glutathione peroxidase (GPx), and total antioxidant status (TAS), were analyzed in brain homogenates. **Results:** Behavioral analyses indicated dose-dependent effects, with higher doses generally reducing activity. MitoCur at 0.25 µM enhanced antioxidant defenses and reduced oxidative damage, while higher doses exhibited a pro-oxidant shift. VPA at 0.25 mM improved TAS without significantly altering MDA levels. **Conclusions:** These findings emphasize the importance of dose optimization in antioxidant-based epilepsy treatments and highlight the potential of MitoCur as a targeted therapeutic option.

## 1. Introduction

Epilepsy remains one of the most prevalent and complex neurological disorders, posing significant challenges to global health and therapeutic innovation [1]. Despite advances in neurology and pharmacology, its management continues to encounter major hurdles, particularly in addressing drug resistance and optimizing treatment strategies. The multifaceted nature of epilepsy [2], characterized by diverse etiologies and clinical manifestations, highlights the need for integrative research approaches spanning molecular, oxidative, and behavioral domains [3,4,5,6].

A critical aspect of epilepsy pathology involves oxidative stress, a key driver of seizure development and progression [7,8,9]. Oxidative stress arises from an imbalance between reactive oxygen species (ROS) [10,11,12] production and the antioxidant defense system, ultimately contributing to neuronal damage, mitochondrial dysfunction, and neuroinflammation [13,14]. This imbalance intensifies neuronal excitability and increases seizure susceptibility, creating a cycle that accelerates epileptogenesis [15].

While it is well established that seizures can generate oxidative stress, increasing evidence suggests that oxidative stress can also act as a contributing factor to seizure onset and progression [8]. Studies have demonstrated that oxidative stress can lower the seizure threshold and promote epileptogenesis through mechanisms such as mitochondrial dysfunction, excitotoxicity, and neuroinflammation [7,16]. Additionally, experimental models have shown that pro-oxidant conditions, such as genetic deletion of antioxidant enzymes, can lead to increased susceptibility to seizures [17,18]. There are also models where seizures can be directly induced by increasing oxidative stress. For example, exposure to pro-oxidant compounds such as kainic acid and pilocarpine has been shown to elevate ROS levels, leading to seizure activity [19].

Our study focuses on biochemical markers such as malondialdehyde (MDA), superoxide dismutase (SOD), glutathione peroxidase (GPx), and total antioxidant status (TAS), which provide insight into the oxidative stress response. Elevated MDA reflects lipid peroxidation and oxidative damage [20], while increased SOD and GPx activities indicate enhanced antioxidant defenses [21,22]. TAS represents the overall antioxidant capacity, with reductions suggesting oxidative imbalance [23].

Analyzing behavioral parameters is crucial for understanding the neurological and pharmacological effects of tested compounds, particularly in models of epilepsy [24]. Behavioral assessments provide a direct, observable measure of how a compound influences brain function, offering insights into locomotion, spatial exploration, and anxiety-like behaviors [24,25]. Parameters like time spent in the top vs. bottom zones can reflect anxiety-like behavior, with increased top-dwelling indicating anxiolysis. Zone transitions and rotational behaviors reflect exploratory activity and neurological status, while movement patterns, distance traveled, and velocity provide insights into locomotor function, with reductions often associated with sedation or motor impairment [24,26,27,28,29]. In epilepsy research, these metrics are especially valuable as they can reflect subtle changes in neural excitability, drug efficacy, and seizure susceptibility [25,30,31]. By analyzing these behaviors prior to seizure induction, researchers can differentiate baseline drug effects from those influenced by seizures, enabling a more comprehensive understanding of a compound’s therapeutic profile [32,33,34,35]. Furthermore, these behavioral endpoints serve as translational markers, as they often parallel symptoms observed in human epilepsy, making them essential for bridging preclinical findings with potential clinical applications [36,37,38].

Curcumin (Cur), a polyphenolic compound derived from *Curcuma longa* L., has gained attention for its antioxidant and anti-inflammatory properties [39,40,41], with emerging evidence suggesting neuroprotective effects in preclinical epilepsy models. However, poor solubility and bioavailability limit its therapeutic potential [42,43,44,45], prompting the development of derivatives such as mitocurcumin (MitoCur) [46,47,48,49,50]. By leveraging triphenylphosphonium (TPP) moieties that selectively accumulate in mitochondria, MitoCur aims to enhance antioxidant activity precisely where ROS are predominantly generated [51]. Meanwhile, sodium valproate (VPA), a well-established antiepileptic drug, also modulates oxidative stress pathways and inflammatory cascades, although its influence on redox balance can vary depending on dose and regimen [52,53].

The zebrafish (*Danio rerio*) has emerged as a powerful model organism for epilepsy research due to its genetic similarity to humans, cost-effectiveness, and amenability to real-time behavioral analyses [54,55,56]. Zebrafish models enable the quantification of various behavioral parameters, such as locomotion (velocity, distance moved, and time spent moving), spatial exploration (time spent in specific zones and transitions between them), and directional movement (clockwise vs. counterclockwise rotations) [25,33,57,58]. Evaluating these metrics prior to and during pentylenetetrazole (PTZ)-induced seizures offers a systematic platform for assessing anticonvulsant efficacy and seizure-associated behaviors in a manner that parallels human epilepsy.

Building on preliminary findings that MitoCur outperforms both Cur and VPA in mitigating seizure severity in the zebrafish PTZ model, this study further investigates the activity of these compounds in the zebrafish model, contributing to a deeper understanding of their effects on behavior, oxidative stress, and their potential therapeutic relevance.

## 2. Results

A range of behavioral parameters—locomotion (time spent moving), spatial exploration (time spent in specific zones and transitions between them), and directional movement (clockwise vs. counterclockwise rotations)—were systematically investigated to assess the effects of the tested compounds prior to PTZ exposure. These behavioral metrics were further complemented by an analysis of oxidative stress parameters, such as SOD, MDA, GPx, and TAS, to explore the relationship between oxidative stress and seizure-related behaviors. To evaluate both behavioral and oxidative stress parameters, we investigated the effects of Cur at 0.25 µM and 0.5 µM, VPA at 0.25 mM and 0.5 mM, and MitoCur at 0.25 µM and 0.5 µM, aiming to explore dose-dependent effects.

### 2.1. Behavioral Parameters

The data were analyzed using one-way ANOVA, followed by Tukey’s post hoc test for multiple comparisons, with a significance level set at α = 0.05. Results are presented as the mean ± standard deviation, highlighting significant differences compared to the control or between treatment groups.

**Figure 1:**
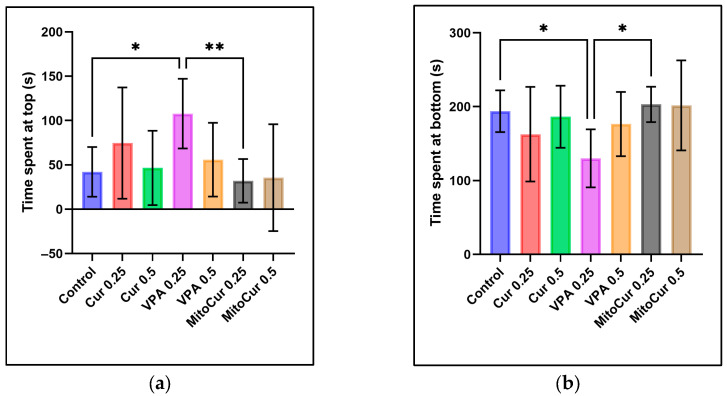
Time spent in different zones of the tank (mean ± SD): (**a**) Time spent at the top (seconds): VPA 0.25 mM significantly increased the time spent at the top compared to the control (*p* = 0.031) and MitoCur 0.25 μM (*p* = 0.008); (**b**) time spent at the bottom (seconds): VPA 0.25 mM significantly decreased the time spent at the bottom compared to the control (*p* = 0.044) and MitoCur 0.25 μM (*p* = 0.013). One-way ANOVA followed by Tukey’s post hoc test (α = 0.05) with * *p* < 0.05, ** *p* < 0.01 indicating statistical significance.

The analysis of time spent at the top (Figure 1a) revealed that VPA 0.25 mM significantly increased the time spent at the top compared to the control group (*p* = 0.031) and compared to MitoCur 0.25 μM (*p* = 0.008). No significant differences were observed between the control group and other treatments, including Cur and MitoCur at either dose. Similarly, the analysis of the time spent at the bottom (Figure 1b) revealed significant differences between VPA 0.25 mM and the control group, with VPA significantly reducing the time spent at the bottom (*p* = 0.044) and also showing a significant decrease compared to MitoCur 0.25 μM (*p* = 0.013). No significant differences were observed between the control group and other treatments, including Cur and MitoCur at either dose.

**Figure 2:**
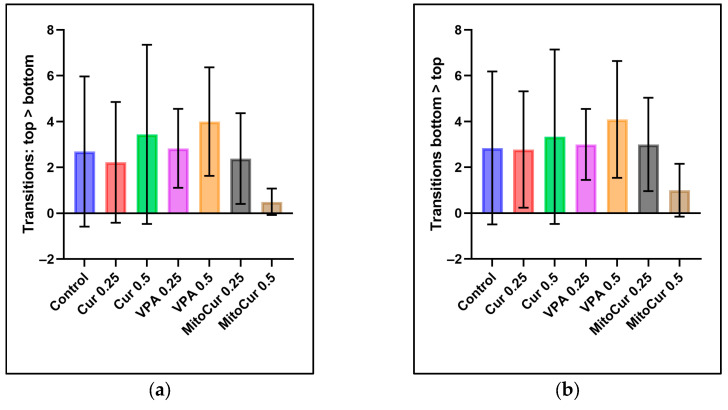
Number of transitions between different zones of the tank (mean ± SD): (**a**) Transitions from top to bottom: no statistically significant differences were observed between the control group and any treatment group; (**b**) transitions from bottom to top: similarly, no statistically significant differences were observed. One-way ANOVA followed by Tukey’s post hoc test (α = 0.05).

The analysis of transitions from top to bottom (Figure 2a), or from bottom to top (Figure 2b) revealed no statistically significant differences between the control group and any of the treatment groups. Similarly, no significant differences were observed between any pair of treatment groups.

**Figure 3:**
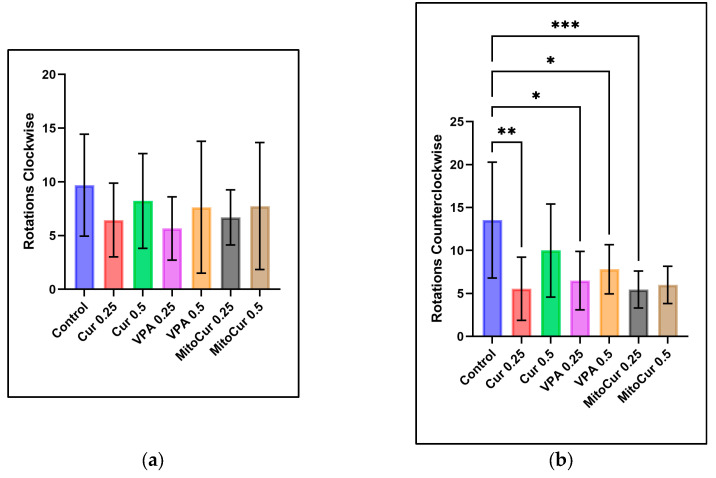
Number of rotations (mean ± SD): (**a**) Clockwise rotations: No statistically significant differences were observed between the control group and any treatment group. (**b**) Counterclockwise rotations: Significant reductions were observed for Cur 0.25 μM (*p* = 0.002), VPA 0.25 mM (*p* = 0.027), VPA 0.5 mM (*p* = 0.033), and MitoCur 0.25 μM (*p* < 0.001) compared to the control. MitoCur 0.5 μM did not show significant differences compared to the control. No significant differences were observed among the treatment groups. One-way ANOVA followed by Tukey’s post hoc test (α = 0.05) with * *p* < 0.05, ** *p* < 0.01, and *** *p* < 0.001 indicating statistical significance.

The results indicated no statistically significant differences in the number of clockwise rotations (Figure 3a) among any of the treatment groups compared to the control group, nor were any significant differences observed between treatment groups. However, the analysis of counterclockwise rotations (Figure 3b) revealed significant reductions in several treatment groups compared to the control. Specifically, Cur 0.25 μM significantly reduced counterclockwise rotations (*p* = 0.002), as did VPA 0.25 mM (*p* = 0.027), VPA 0.5 mM (*p* = 0.033), and MitoCur 0.25 μM (*p* < 0.001). Neither Cur 0.5 μM nor MitoCur 0.5 μM showed statistically significant differences compared to the control group, and no significant differences were observed among the treatment groups.

**Figure 4:**
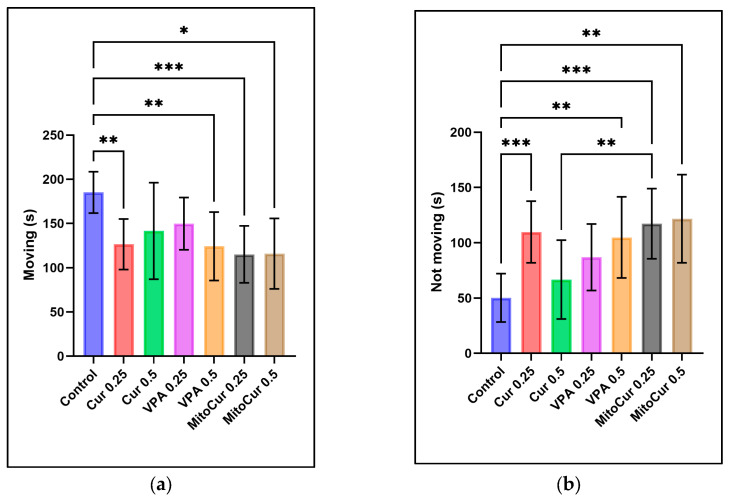
Locomotor activity (mean ± SD): (**a**) Time spent moving (seconds): The control group spent significantly more time moving compared to Cur 0.25 μM (*p* < 0.01), VPA 0.5 mM (*p* < 0.001), MitoCur 0.25 μM (*p* < 0.001), and MitoCur 0.5 μM (*p* < 0.01). (**b**) Time spent not moving (seconds): Cur 0.25 μM (*p* < 0.001), VPA 0.5 mM (*p* < 0.01), MitoCur 0.25 μM (*p* < 0.001), and MitoCur 0.5 μM (*p* < 0.01) significantly increased immobility compared to the control. One-way ANOVA followed by Tukey’s post hoc test (α = 0.05) with * *p* < 0.05, ** *p* < 0.01, and *** *p* < 0.001 indicating statistical significance.

The analysis of moving time (Figure 4a) revealed significant differences between the control group and several treatment groups. The control group spent significantly more time moving compared to Cur 0.25 μM (*p* = 0.006), VPA 0.5 mM (*p* = 0.002), and both doses of MitoCur 0.25 μM (*p* < 0.001) and 0.5 μM (*p* = 0.019). No significant differences were observed between the control group and Cur 0.5 μM or VPA 0.25 mM. For immobility (Figure 4b), significant increases were observed in certain treatment groups compared to the control. Cur 0.25 μM significantly increased the time spent not moving (*p* < 0.001), as did VPA 0.5 mM (*p* = 0.001). Both doses of MitoCur significantly increased immobility: MitoCur 0.25 μM (*p* < 0.001) and MitoCur 0.5 μM (*p* = 0.003). Additionally, a significant difference was observed between Cur 0.5 μM and MitoCur 0.25 μM (*p* = 0.008), highlighting a stronger impact of MitoCur 0.25 μM on immobility. No significant differences were observed for Cur 0.5 μM or VPA 0.25 mM compared to the control.

### 2.2. Oxidative Stress Parameters

**Figure 5:**
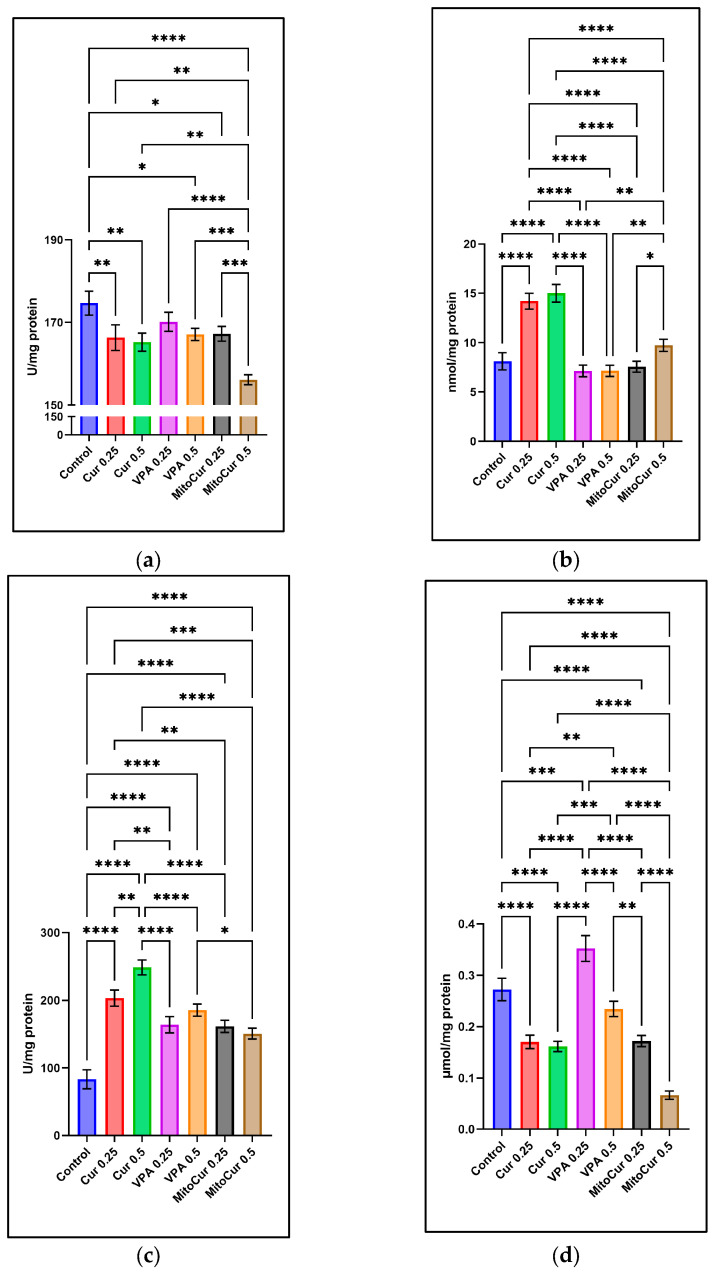
Effects of Cur, VPA, and MitoCur on oxidative stress and antioxidant markers (Mean ± SD): (**a**) SOD. The control group exhibited the highest SOD activity, which was significantly reduced by all treatments except VPA 0.25 mM. (**b**) MDA. MDA levels were elevated by Cur 0.25 µM and 0.5 µM and MitoCur 0.5 µM, while VPA 0.25 mM and 0.5 mM and MitoCur 0.25 µM showed no significant changes. (**c**) GPx. GPx activity was significantly increased across all treatments compared to Control, indicating a compensatory antioxidant response. (**d**) TAS. TAS was notably elevated by VPA 0.25 mM, while Cur 0.25 µM and 0.5 µM and MitoCur 0.25 µM and 0.5 µM significantly reduced TAS, suggesting a depletion of overall antioxidant capacity. One-way ANOVA followed by Tukey’s post hoc test (α = 0.05) with * *p* < 0.05, ** *p* < 0.01, *** *p* < 0.001 and **** *p* < 0.0001 indicating statistical significance.

The parameters analyzed included SOD (Figure 5a), MDA (Figure 5b), GPx (Figure 5c), and TAS (Figure 5d), providing a detailed assessment of each compound’s impact on oxidative stress and antioxidant defense systems.

SOD activity (Figure 5a) was significantly higher in the control group compared to most treatment groups. Specifically, SOD activity in the control group was significantly greater than for Cur 0.25 µM (*p* < 0.01), Cur 0.5 µM (*p* < 0.01), VPA 0.5 mM (*p* < 0.05), MitoCur 0.25 µM (*p* < 0.05), and MitoCur 0.5 µM (*p* < 0.0001). VPA 0.25 mM was the only treatment not significantly different from the control (*p* > 0.05). MitoCur 0.5 µM elicited the strongest reduction in SOD. These findings suggest that while the control group maintained higher SOD activity, Cur, higher dose of VPA, and MitoCur reduced SOD levels, potentially altering enzymatic antioxidant defense.

MDA (Figure 5b), an indicator of lipid peroxidation, was significantly elevated in some treatments. Cur 0.25 µM and 0.5 µM both significantly increased MDA levels (*p* < 0.0001), indicating heightened oxidative stress. MitoCur 0.5 µM also significantly increased MDA compared to the control (*p* < 0.01). In contrast, VPA at both 0.25 mM and 0.5 mM and MitoCur 0.25 µM did not show significant changes in MDA (*p* > 0.05). These results suggest that while higher doses of Cur and MitoCur exacerbate lipid peroxidation, lower-dose MitoCur and VPA do not significantly affect this marker of oxidative stress.

GPx activity (Figure 5c) was significantly increased across all treatments compared to the control (*p* < 0.0001 for most comparisons), indicating a universal adaptive response to oxidative stress. Among the treatments, Cur 0.5 µM showed the highest increase in GPx activity, followed closely by VPA 0.25 mM and 0.5 mM. MitoCur at both doses also elevated GPx activity, although to a slightly lesser extent. These results highlight the potential of these compounds to robustly enhance antioxidant defenses in a dose-dependent manner.

Distinct patterns were observed for TAS (Figure 5d). VPA 0.25 mM significantly increased TAS compared to the control (*p* < 0.001), indicating enhanced overall antioxidant capacity. Conversely, Cur and MitoCur at both doses (0.25 µM and 0.5 µM) significantly reduced TAS (all *p* < 0.0001), indicating a net depletion of antioxidant reserves. VPA 0.5 mM showed no significant change in TAS (*p* > 0.05).

These findings reveal the nuanced and dose-dependent impacts of the tested compounds on oxidative stress markers, highlighting the need for careful dose optimization to achieve therapeutic benefits while minimizing oxidative damage.

## 3. Discussion

Behavioral and oxidative stress analyses are essential for understanding the pharmacological effects of tested compounds in epilepsy models. Behavioral parameters provide insights into locomotor activity and potential neuromodulatory effects, while oxidative stress markers help assess the balance between antioxidant defenses and oxidative damage. Evaluating these factors before seizure induction allows for a clearer distinction between baseline drug effects and seizure-related changes, offering valuable translational insights for potential clinical applications.

A preference for the lower portion of the tank is commonly interpreted as heightened anxiety-like behavior in zebrafish, whereas spending more time in the upper regions typically indicates anxiolysis [59,60,61]. We observed that VPA 0.25 mM significantly increased the time spent in the top zone while reducing the time in the bottom zone compared to controls and MitoCur 0.25 µM, suggesting an anxiolytic-like effect at this lower VPA dose. In contrast, neither Cur (0.25 µM or 0.5 µM) nor MitoCur (0.25 µM or 0.5 µM) significantly changed the vertical distribution of zebrafish. These findings imply that, under our specific dosing and exposure conditions, VPA also modulated anxiety-like behavior. Various pharmacological compounds—such as diazepam, buspirone, and ethanol (in certain doses)—were shown to reduce bottom-dwelling, reinforcing the notion that increased top-dwelling may reflect reduced anxiety [62,63]. VPA’s known enhancement of GABAergic neurotransmission [64] could explain why it effectively shifted zebrafish to higher zones in our study, whereas Cur has produced mixed results in other models [65], possibly due to dose or bioavailability factors.

Despite VPA 0.25 mM affecting time spent in the top zone, none of the treatments significantly altered the frequency of transitions between the top and bottom zones. Thus, VPA’s action at 0.25 mM appears to specifically reduce bottom-dwelling rather than broadly altering exploratory movements or hyperactivity. Transitions between vertical zones can be indicative of either anxiety-like or hyperactive states [24]. In some zebrafish studies, compounds like MK-801, a pore blocker of the NMDA receptor, decrease bottom-dwelling (suggesting anxiolysis) but can simultaneously increase locomotion, which complicates interpretations of drug effects [66,67]. Each subject had a minute to familiarize itself with the tank, trying to minimize the novelty-induced bottom-dwelling. Over longer exposures, zebrafish often habituate and reduce bottom-dwelling behavior [68,69].

We found that while clockwise rotations remained unchanged, counterclockwise (CCW) rotations significantly decreased under Cur 0.25 µM, VPA 0.25 mM and 0.5 mM, and MitoCur 0.25 µM. Interestingly, MitoCur 0.5 µM did not replicate this reduction. Because CCW rotations are widely considered markers of abnormal swimming and heightened anxiety-like behavior, these decreases could signify reduced stereotypic or anxiety-like responses. Previous research has shown that elevated CCW rotations can be linked to stress or toxicity in zebrafish [70]. For instance, Robea et al. noted increased CCW rotations when zebrafish were exposed to Fipronil and Pyriproxyfen, indicating heightened anxiety [71]. In rodent stroke models, unilateral neurological damage leads to more CCW rotations, illustrating how lateralized motor deficits can also manifest as asymmetrical circling [72]. Taken together, our decreases in CCW rotations under Cur, VPA, and MitoCur point toward a calming effect, although further work is needed to clarify the neural circuitry underlying rotational behavior [24].

Cur 0.25 µM, VPA 0.5 mM, and MitoCur at both 0.25 µM and 0.5 µM significantly decreased overall moving time and increased immobility compared to control. However, Cur 0.5 µM and VPA 0.25 mM did not produce a notable reduction in locomotion, implying a dose-dependent difference. While decreased locomotion can reflect mild sedation if zebrafish adopt a calmer behavioral state [57], curcumin is known to interact with dopaminergic and serotonergic pathways [73,74], though its effects can vary with concentration [75]. Distinguishing immobility caused by mild sedation or toxicity from freezing caused by stress or fear is crucial. Zebrafish freezing behavior involves elevated respiration, whereas sedation-induced immobility often presents alongside reduced opercular movements [24]. Higher doses of compounds such as lysergic acid diethylamide (LSD) and chlordiazepoxide, for example, reduce freezing and increase top-dwelling in zebrafish, indicating an anxiolytic rather than sedative profile [76,77].

In our previous study, it was shown that all treatments at either dose tended to reduce total distance moved and velocity compared to the control group, with no strong dose-dependent patterns [50]. This uniformity suggests that the compounds might share baseline motor-suppressive or anxiolytic properties rather than exhibiting simple dose-dependent effects. Distance moved and velocity are critical parameters for assessing zebrafish activity, providing valuable insights into their neurological and physiological states [24]. Previous studies have reported that curcumin can reduce the total distance moved in adult zebrafish, while findings on VPA have been less consistent [57]. These differences may reflect variations in experimental conditions, such as study design, dosing regimens, or zebrafish strain-specific factors, highlighting the complexity of comparing results across different studies. Notably, the observed similarity in locomotor reductions across Cur, VPA, and MitoCur suggests that the anticonvulsant or neuroprotective properties observed following PTZ challenge may be influenced by specific pharmacodynamic mechanisms, rather than being solely attributable to sedation.

PTZ-induced seizures have been shown to exacerbate oxidative stress by increasing lipid peroxidation and depleting antioxidant defenses, such as SOD and GPx, particularly in brain regions like the hippocampus and cerebral cortex [18]. In our study, zebrafish were pre-exposed to Cur, VPA, and MitoCur to mitigate these effects, demonstrating the potential of these compounds to prevent oxidative damage and preserve redox balance.

Our results demonstrated that Cur at higher concentrations significantly elevated MDA levels, reduced TAS, and caused a significant rise in GPx activity, suggesting a paradoxical pro-oxidant behavior under certain experimental conditions. Prior reports indicate that Cur generally exhibits antioxidant and anti-inflammatory properties [78]. Its safety profile at therapeutic doses extends to curcumin-loaded nanocomplexes, which have shown minimal toxicity in rodents and hamsters, with chronic dosing at 0.27–0.54 g/kg body weight/day producing no observable adverse effects [79]. Similarly, curcumin-essential oil complexes administered at a dose of 5000 mg/kg body weight in rats showed no toxicity, genotoxicity, or mutagenicity, supporting Cur’s non-toxic pharmacological profile under controlled dosing regimens [80].

Although Cur is recognized for its antioxidant and anti-inflammatory properties, several factors can drive it toward a pro-oxidant behavior [48,81,82,83,84]. Reports suggest that Cur, in a concentration-dependent manner, plays both antioxidative and prooxidative roles in neuronal tissues. A study on hepatocytes demonstrated a protective effect of low concentrations of Cur by reducing lipid peroxidation and cytochrome c release, while opposite effects were observed through caspase-3 activation at higher concentrations [85,86]. Additional contributors to Cur’s pro-oxidant activity include its α,β-unsaturated carbonyl moiety, which can generate ROS [87], and its ability to bind transition metals (e.g., copper, iron), further amplifying oxidative stress [88]. Moreover, interactions with the thioredoxin system also play a critical role in its dose-dependent redox effects. Cur can modify thioredoxin reductase, converting it from an antioxidant enzyme into a ROS generator by channeling electrons from NADPH to oxygen [89]. Interestingly, in vitro studies demonstrate that N-acetylcysteine can partially reverse Cur-induced apoptosis by quenching excess ROS, highlighting the delicate balance between Cur’s antioxidant and prooxidant activities [90].

In our study, MitoCur also showed a dose-dependent effect. At lower doses (0.25 µM), MitoCur effectively reduced MDA levels—a marker of lipid peroxidation—while modestly increasing GPx activity. These findings point toward enhanced antioxidant defenses without overwhelming cellular systems. Conversely, at higher doses (0.5 µM), MitoCur shifted toward a pro-oxidant profile, evidenced by decreased SOD activity and TAS.

MitoCur is known for its enhanced mitochondrial targeting. Triphenylphosphonium—a lipophilic cation—enables selective delivery of MitoCur to mitochondria, achieving up to 1000-fold higher local concentrations [91,92]. Therefore, by rapidly modulating oxidative pathways at the mitochondrial level, MitoCur could stabilize mitochondrial function. Parallel mechanisms are seen in other mitocans like MitoQ, which can reduce lipid peroxidation and facilitate brain absorption by increasing mitochondrial membrane permeability, thus offering neuroprotection in diseases with oxidative-stress-driven cell death [93,94]. Similar to SkQ1, which is an antioxidant at low concentrations but prooxidant at higher levels, MitoQ generally does not overpower the body’s antioxidant systems [95]. Preliminary tests in our lab showed MitoCur exerting more pronounced effects than native Cur at the same dose. Nevertheless, the redox cycling of MitoCur follows a pattern where higher doses shift to a prooxidant effect that exacerbates oxidative stress—mirroring reports of MitoCur’s prooxidant behaviors under certain conditions [48,51].

It is worth mentioning that MitoCur also interacts with the thioredoxin system. It binds with high affinity to mitochondrial TrxR2, disrupting its activity [46]; at lower doses, such inhibition may temper excessive ROS production, whereas at higher doses, MitoCur-induced inhibition fosters substantial mitochondrial oxidative stress. Additionally, the Nrf2 pathway could mediate MitoCur’s antioxidant response at subtoxic concentrations. Mild increases in ROS may dissociate the Keap1–Nrf2 complex, allowing Nrf2 to enter the nucleus and induce genes such as heme oxygenase-1 (HO-1), SOD2, and GPx [96]. This may stabilize mitochondrial function and enhance cellular resilience. However, robust ROS elevations at higher doses might overwhelm these protective responses, tilting the balance toward oxidative injury. Consequently, a detailed dose–response analysis spanning lower to higher doses is essential for MitoCur, avoiding thresholds at which its effects become detrimental.

VPA exhibited a distinct oxidative stress profile. At a lower dose (0.25 µM), VPA increased TAS without elevating MDA, suggesting an ability to boost endogenous antioxidant defenses. A study investigating PTZ-induced seizures in mice demonstrated that VPA reduced oxidative stress markers (MDA), increased antioxidant enzyme activities (SOD and GPx), decreased neuronal damage, and attenuated inflammation [97]. Supporting this, in a glutamate-induced excitotoxicity in vitro model, VPA pre-treatment reduced oxidative damage by lowering MDA and H_2_O_2_ levels, thereby enhancing cell viability, though its effects on antioxidant enzymes like CAT and SOD were limited [53]. Another study on EoL-1 cells demonstrated that VPA has the potential to induce ROS and impact cell viability by suppressing the negative regulator of Nrf2, a key transcription factor that activates antioxidant defenses [52]. Simultaneously, VPA has been shown to increase autophagic markers and the expression of NLRP3 and NLRC4 mRNA, highlighting its role in modulating autophagy. Furthermore, VPA can regulate inflammatory responses through dose-dependent effects on inflammasome-driven caspase-1 deactivation, underscoring its complex interplay between oxidative stress, autophagy, and inflammation [52,98]. Overall, our findings suggest complex and dose-dependent effects of Cur, VPA, and MitoCur on zebrafish behavioral outcomes and oxidative status. VPA at a lower dose exhibited notable effects on top-dwelling and an increase in TAS, suggesting a milder behavioral modulation potentially linked to balanced redox responses. Whereas our earlier work established MitoCur’s potential to delay seizure onset and reduce seizure severity, these new data show that MitoCur can also influence locomotor activity and redox homeostasis in a dose-dependent manner: lower doses curtailed certain parameters of motor activity and MDA, whereas higher doses tended toward pro-oxidant effects and reduced TAS. These oxidative shifts frequently occurred alongside changes in locomotion and exploration, suggesting that redox fluctuations may affect or mirror the observed behavioral profiles—potentially reflecting changes in arousal levels. The findings underline the importance of the “antioxidant window” concept, wherein MitoCur exhibits antioxidant effects at lower doses but may shift toward prooxidant activity at higher concentrations, emphasizing the need for careful dosage optimization to ensure therapeutic efficacy without triggering oxidative damage. As a potential direction for future research, investigating intracellular ROS levels, mitochondrial function, and apoptosis markers could further elucidate the molecular pathways through which MitoCur exerts its therapeutic effects.

## 4. Materials and Methods

### 4.1. Animals

Adult zebrafish (*Danio rerio*, wild-type AB strain, aged 4–9 months) of both sexes were sourced from a local supplier and acclimatized for four weeks prior to experimentation at the “Ion Ionescu de la Brad” Iași University of Life Sciences, Iași, Romania. To minimize variability in pharmacokinetics and pharmacodynamics, the fish were carefully weighed and measured, ensuring a consistent selection within the range of 250–350 mg in weight and 18.3–25.7 mm in length. They were housed at a density of 60–70 fish per aquarium in 37 L tanks (dimensions: 45 × 28 × 30 cm) filled with dechlorinated water treated with 132 mL/L AquaSafeH (Tetra, Blacksburg, VA, USA). The tanks were maintained at 26 ± 2 °C with continuous filtration and aeration under a 14:10 h light/dark cycle (lights on at 8:00 a.m. and off at 10:00 p.m.), and the water’s pH was monitored regularly to remain between 7.0 and 7.25 using pH strips. Fish were fed twice daily with a standard semi-floating aquarium feed (42% protein, min 4% fat, max 3% fiber, max 12% ash, non-colored).

All zebrafish were healthy, experimentally naive, and exhibited no signs of disease. Randomized selection was performed without consideration of sex. Testing occurred during the light phase, specifically between 8:00 a.m. and 2:00 p.m., following a minimum acclimation period of 30 min in the experimental room.

At the end of the experiments, the zebrafish were euthanized by immersion in an ice-water bath (5 parts ice to 1 part water, temperature range 0–4 °C). All experimental procedures adhered to the European Union Directive 2010/63/EU (dated 22 September 2010) and Romanian regulations for animal experimentation. Approval for the study protocols was obtained from the Local Ethics Committee of the “Ion Ionescu de la Brad” Iași University of Life Sciences (license no 1760/24 October 2023). The study design strictly followed the principles of replacement, reduction, and refinement (3Rs), ensuring the use of the smallest possible number of animals while maintaining statistical reliability. Every effort was taken to reduce animal discomfort throughout the study.

### 4.2. Chemicals and Reagents

**Figure 6:**
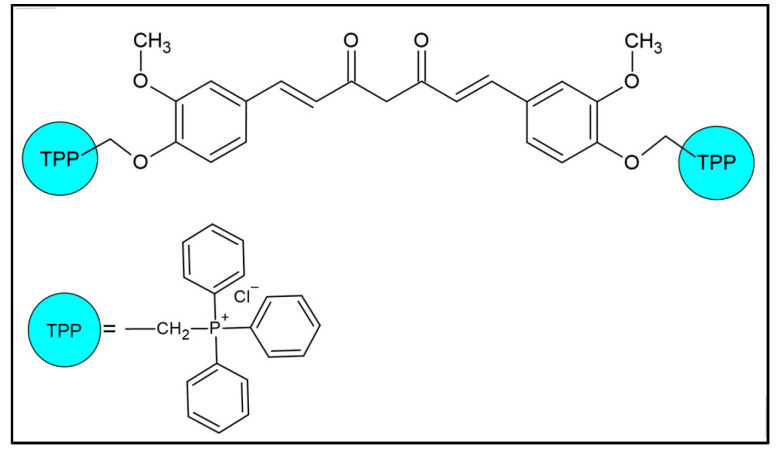
The structure of the mitocurcumin used in the present study. TPP = triphenylphosphonium. Generated with ChemSketch (Freeware) 2024.1.3.

Curcumin, pentylenetetrazole, and sodium valproate were procured from Sigma-Aldrich (St. Louis, MO, USA). Mitocurcumin (1,7-Bis{3-methoxy-4-[3-(triphenylphosphonium)propoxy]-phenyl} hepta-1,6-diene-3,5-dione dichloride), as shown in Figure 6, was sourced from Chiralsyn Laboratories (Hyderabad, India). All additional reagents utilized were of analytical grade.

Cur and MitoCur were dissolved in 0.1% anhydrous dimethyl sulfoxide (DMSO) from Sigma-Aldrich and subsequently diluted in the system water. Sodium valproate was prepared using ultrapure water, while PTZ was dissolved directly in aquarium water. All solutions were freshly prepared prior to use.

### 4.3. Experiment Design

The animals were exposed by immersion to the tested compounds at two concentrations for a duration of 30 min. Following this exposure, the fish were observed for 5 min to evaluate the behavioral effects of the compounds. To examine the antiepileptic properties of the tested compounds in PTZ-induced seizures, all groups were subsequently immersed in PTZ for 10 min, during which their seizure behavior was monitored. At the conclusion of the experiment, the animals were euthanized, and the concentrations of the compounds in their brains were measured.

**Figure 7:**
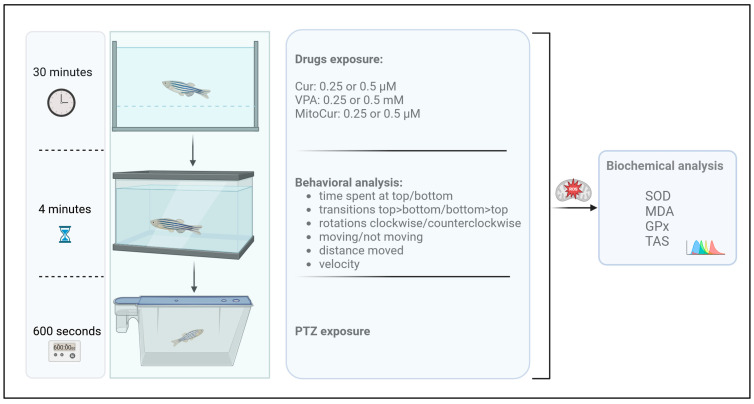
Schematic illustration of the experimental workflow. Adult zebrafish were immersed for 30 min in one of three compound solutions—Cur (0.25 µM or 0.5 µM), VPA (0.25 mM or 0.5 mM), or MitoCur (0.25 µM or 0.5 µM). Immediately afterward, each fish was placed in a test tank for 4 min to assess baseline locomotor and exploratory parameters (e.g., time spent in top vs. bottom, rotational movements, transitions, distance moved, and velocity). Next, the animals were transferred to a separate tank containing PTZ (5 mM) for 10 min (600 s) to induce and observe seizure activity. Following these behavioral assessments, the fish were euthanized, and brain tissues were collected for biochemical analyses of oxidative stress and antioxidant defense markers, including SOD, MDA, GPx, and TAS.

The experimental setup (Figure 7) consisted of a control group (*n* = 15) that did not receive any antiepileptic treatment and six treatment groups. These included exposure to 0.25 µM (*n* = 14) and 0.5 µM (*n* = 10) Cur, 0.25 µM (*n* = 10) and 0.5 µM (*n* = 16) MitoCur, and 0.25 mM (*n* = 8) and 0.5 mM VPA (*n* = 13). VPA served as a reference to validate the zebrafish response to a traditional antiepileptic drug in the PTZ seizure model.

Each treated animal was individually immersed in a beaker containing 0.5 L of the respective compound solution for 30 min prior to PTZ exposure. All groups underwent identical treatment and analysis procedures.

The selected concentrations for Cur (0.25 µM and 0.5 µM) and VPA (0.25 mM and 0.5 mM) were informed by existing literature [33,57,99] and preliminary laboratory studies. Likewise, the concentrations of MitoCur (0.25 µM and 0.5 µM) were chosen to align with Cur doses and supported by findings from our initial experiments.

To induce clonus-like epileptic seizures, each fish was individually immersed in 5 mM PTZ, dissolved in water. The PTZ seizure-inducing protocol, including concentration and exposure duration, was based on prior studies [33,57,100,101,102,103]. Immediately following the initial behavioral activity assessment, the animals were transferred into 500 mL beakers containing the 5 mM PTZ solution.

### 4.4. Behavioral Assessment—Locomotor and Exploratory Behavior

Following the antiepileptic pre-treatment, each fish was gently placed in an individual rectangular tank (18 × 13 × 15 cm) filled with 2.25 L of system water. Behavioral activity was recorded for a 5 min session using a digital webcam connected to a computer, positioned 40 cm from the tank to ensure the apparatus was fully within the camera’s field of view [104]. To evaluate the effects of the tested compounds on locomotor and exploratory behavior, EthoVision XT version 16 video tracking software (Noldus Information Technology, Wageningen, The Netherlands) was utilized to analyze zebrafish tank exploration [77]. The first minute was designated for habituation to the apparatus, after which behavioral activity was recorded for the remaining 4 min. Locomotion data, including distance moved and velocity, were subsequently analyzed as key endpoints [105].

**Figure 8:**
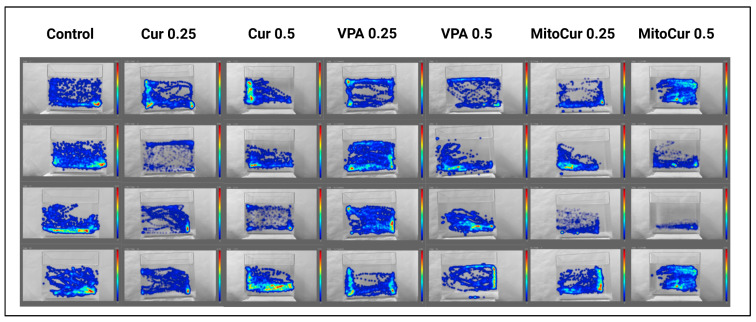
Examples of behavioral patterns and swimming paths; trends in behavioral responses. The 2D heat maps illustrate the spatial distribution of zebrafish swimming behavior across treatment groups for the YZ axis (front view). This visualization demonstrates changes in locomotor and exploratory behaviors across the tested conditions, reflecting compound-specific and dose-dependent effects on zebrafish activity prior to PTZ-induced seizures.

Figure 8 provides a visual representation of zebrafish swimming behavior under different treatment conditions, illustrating spatial activity patterns using 2D heat maps (YZ axis, front view).

These heat maps highlight variations in locomotor and exploratory behavior across treatment groups, with warmer colors indicating higher activity levels. The figure showcases how the dose-dependent and compound-specific effects of Cur, VPA, and MitoCur on zebrafish movement were assessed to obtain a comparative overview of behavioral responses prior to PTZ-induced seizures.

The animals were then exposed to PTZ treatment until either reaching the final scoring point, corresponding to tonic seizure-like behavior in zebrafish, or until 600 s had elapsed. All experiments were conducted in triplicate on separate days in a quiet room.

Stringent measures were taken to ensure the reliability of behavioral results while minimizing handling stress. Fish were gently transferred between home tanks, beakers, and experimental setups. Throughout the experiments, all fish were handled consistently, and behaviors were recorded in the same controlled room, ensuring uniformity in manipulation, water quality, and lighting conditions across trials [67].

### 4.5. Biochemical Analyses of Oxidative Stress

All the samples were homogenized (1:9, w: v) in saline solution (0.9%, NaCl) at 4 °C using an ultrasonic homogenizer, and homogenates were centrifuged at 5000× *g* for 15 min at 4 °C. The supernatants were placed in new tubes and kept at −20 °C until the biochemical determinations. Protein measurements in homogenates were performed using the Folin–Ciocalteu’s phenol reagent from Sigma-Aldrich (Merck KGaA, Darmstadt, Germany), using bovine albumin as standard. SOD levels from the samples were analyzed with a Ransod kit from Randox (Crumlin, UK). The method uses a generator of superoxide radicals (xanthine and xanthine oxidase) and a detector of superoxide radicals, the (2-(4-iodophenyl)-3-(4-nitrophenol)-5-phenyltetrazolium chloride (INT). One unit of SOD induces a 50% inhibition of the INT reduction rate. MDA (as the catabolite of lipid peroxides) was determined using a colorimetric kit from ThermoFisher Scientific (Waltham, MA, USA). MDA from samples reacts with thiobarbituric acid (TBA) to produce a red compound with a maximum absorption peak at 532 nm. GPx was measured using a Randox kit (RANSEL from Crumlin, UK). GPx oxidizes reduced glutathione (GSH) in the presence of cumene hydroperoxide. Glutathione Reductase and NADPH convert the oxidized glutathione (GSSG) to the reduced form with a concomitant oxidation of NADPH to NADP^+^, leading to a decrease in absorbance at 340 nm. The TAS assay was performed using a Randox kit (Crumlin, UK), based on the colorimetric measurement of ABTS+ radical (2,2′-Azino-di-[3-ethylbenzthiazoline sulphonate]) which is oxidized to a stable blue-green radical. The antioxidants in the samples inhibit this oxidation.

### 4.6. Data Analysis

Statistical analysis was conducted using GraphPad Prism version 10.3.1 for Windows (GraphPad Software, Boston, MA, USA, www.graphpad.com). The *p* values were adjusted for multiple comparisons.

## 5. Conclusions

In summary, the study demonstrates that MitoCur exerts dose-dependent effects on oxidative stress parameters in the PTZ zebrafish model. At a therapeutically favorable dose of 0.25 μM, MitoCur enhances antioxidant defenses and mitigates oxidative stress, showing efficacy comparable to VPA. These results suggest that MitoCur holds promise as a novel antioxidant therapy in epilepsy treatment. Nevertheless, the potential pro-oxidant effects at higher doses emphasize the importance of dose optimization and further research. Future studies should focus on elucidating the precise mechanisms underlying MitoCur’s actions, exploring its long-term effects, and assessing its efficacy in mammalian models to pave the way for clinical applications in epilepsy management.

## Data Availability

The raw data supporting the conclusions of this article will be made available by the authors on request.

## Abbreviations

The following abbreviations are used in this manuscript:

CAT	Catalase
CCW	Counterclockwise
Cur	Curcumin
DMSO	Dimethyl sulfoxide
GPx	Glutathione peroxidase
LSD	Lysergic acid diethylamide
MDA	Malondialdehyde
MitoCur	Mitocurcumin
PTZ	Pentylenetetrazole
ROS	Reactive oxygen species
SOD	Superoxide dismutase
TAS	Total antioxidant status
TPP	Triphenylphosphonium
VPA	Sodium valproate
